# Hematological parameters as predictors of prostate cancer: A study based on multivariable and bidirectional Mendelian randomization

**DOI:** 10.1097/MD.0000000000044775

**Published:** 2025-09-26

**Authors:** Yirui Wei, Hao Wang, Liang Wang, Pushen Yang, Dawei Xie, Weifeng He, Jianwen Wang

**Affiliations:** a Department of Urology, Beijing Chaoyang Hospital, Capital Medical University, Beijing, China; b Department of TCM Oncology, Beijing Shijitan Hospital, Capital Medical University, Beijing, China.

**Keywords:** hematological parameters, Mendelian randomization, prostate cancer, single nucleotide polymorphisms

## Abstract

This study explores the causal relationship between hematologic parameters and prostate cancer (PCa) risk, offering insights into potential biological pathways and clinical implications. We used genome-wide association studies to perform forward and reverse Mendelian randomization (MR) within a 2-sample framework. Additionally, multivariable MR was employed to assess the associations between multiple hematological markers and PCa. Several sensitivity analyses were conducted to ensure the robustness of the findings. Univariable MR analysis showed that genetically predicted mean corpuscular volume (OR: 0.942, 95% CI: 0.891–0.996, *P* = .035) and mean corpuscular hemoglobin (OR: 0.934, 95% CI: 0.882–0.988, *P* = .018) were associated with reduced PCa risk. Conversely, increases in eosinophil (OR: 1.081, 95% CI: 1.005–1.163, *P* = .036) and basophil counts (OR: 1.235, 95% CI: 1.006–1.516, *P* = .044) were linked to higher PCa risk. Multivariable MR highlighted a stronger association between basophil count and PCa (OR: 1.432, 95% CI: 1.028–1.996, *P* = .034). Reverse MR indicated that PCa may increase neutrophil (OR: 1.012, 95% CI: 1.001–1.023, *P* = .031) and red blood cell counts (OR: 1.008, 95% CI: 1.000–1.016, *P* = .042). Our findings reveal causal associations between hematological parameters and PCa, improving understanding of the genetic links and potential interactions between these markers and PCa risk.

## 1. Introduction

Prostate cancer (PCa) is the second most common malignancy and a leading cause of cancer-related death among men worldwide. In 2023, the United States alone reported approximately 2,88,300 new cases, reflecting the substantial morbidity and mortality associated with the disease.^[1]^ Standard clinical evaluation of PCa involves measuring serum prostate-specific antigen (PSA) levels, imaging studies, and digital rectal examination. However, definitive diagnosis requires histopathological confirmation through transrectal ultrasound-guided prostate biopsy.^[2,3]^

Ongoing research focuses on improving early detection and diagnostic strategies, with a growing emphasis on serum biomarkers.^[4,5]^ Recent studies using UK Biobank data have highlighted associations between hematologic parameters and both the risk and mortality of PCa. Elevated red blood cell (RBC) and platelet counts have been identified as risk factors for PCa, while larger mean corpuscular volume (MCV) and mean corpuscular hemoglobin (MCH) levels are inversely associated with disease risk.^[6]^ RBC distribution width has also emerged as an independent prognostic marker for PCa.^[7]^ Moreover, an elevated neutrophil-to-lymphocyte ratio has been linked to poor prognosis,^[8]^ with neutrophil counts correlating with 5-year survival rates in localized PCa.^[9]^ In metastatic hormone-sensitive PCa, higher baseline basophil counts and basophil-to-lymphocyte ratios have been associated with worse clinical outcomes.^[10]^

These findings suggest that hematologic markers may offer valuable insights into PCa progression and prognosis. However, observational epidemiological studies often face limitations, such as reverse causation, misclassification, and unmeasured confounding, which complicate causal interpretation.^[11]^ Mendelian randomization (MR) has emerged as a robust approach to address these limitations by using genetic variants as instrumental variables (IVs), enabling stronger causal inference.^[12,13]^ Because genetic variations are randomly assigned at conception and unaffected by environmental or lifestyle factors, MR analyses reduce residual confounding and mitigate reverse causation.

In this study, we applied both univariable and multivariable MR analyses across 2 independent cohorts to explore potential causal associations between hematologic parameters and PCa. Our findings aim to clarify the genetic underpinnings of these relationships and provide evidence to support improved follow-up and monitoring protocols. The results may inform targeted interventions and ultimately contribute to better clinical outcomes for individuals with PCa.

## 2. Materials and methods

**Figure 1.**
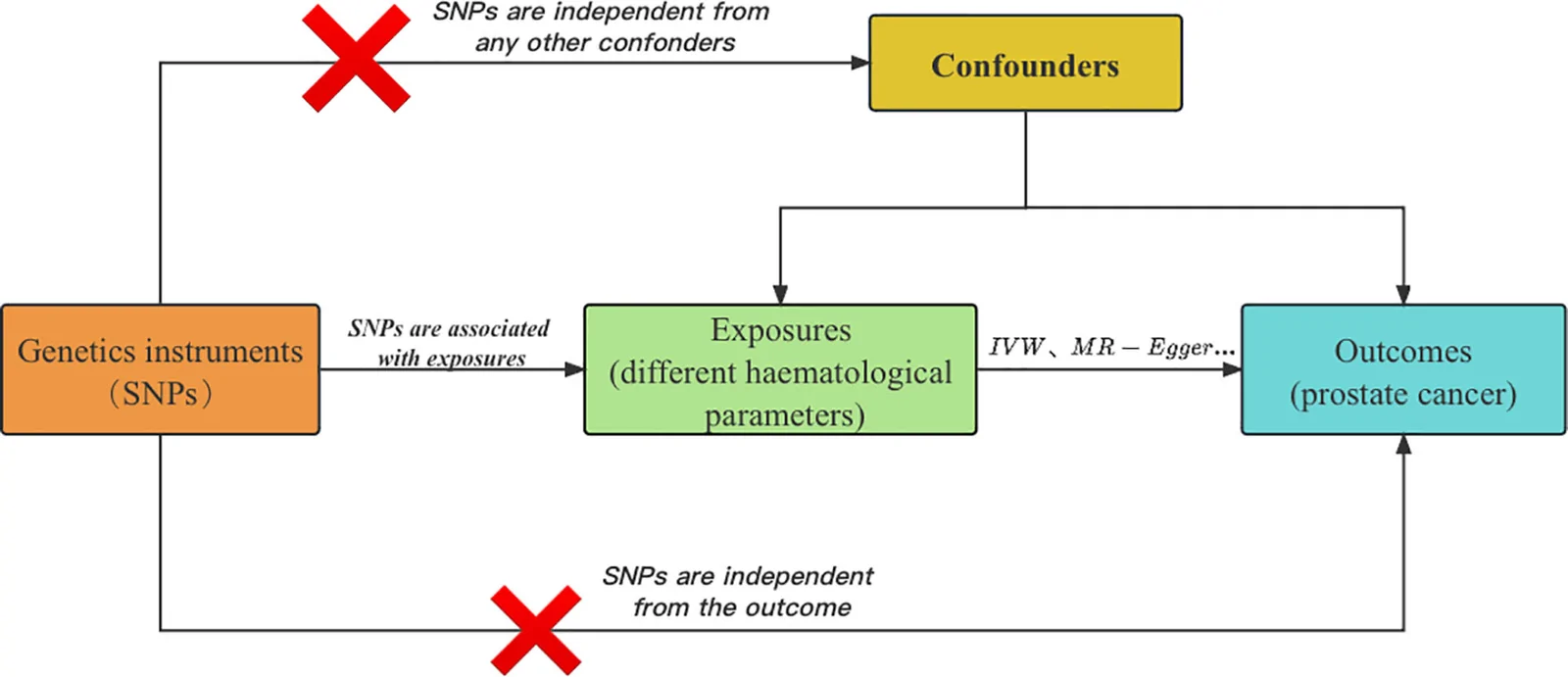
Assumptions underpinning the MR analysis for diverse hematological parameters and PCa. The MR study posits that genetic variants exhibit associations solely with the exposure of interest and are not entangled with confounding factors or alternative causal pathways. IVW = inverse variance weighted, MR = Mendelian randomization, PCa = prostate cancer, SNP = single nucleotide polymorphisms.

Figure 1 illustrates the methodology and core principles of MR employed in this study. Using summary-level data from genome-wide association studies (GWAS), we conducted both univariate (forward and reverse) and multivariate MR analyses within a 2-sample MR framework. MR leverages genetic variants randomly inherited at conception as IVs to infer causal relationships between exposures and outcomes. This approach rests on 3 key assumptions: the selected genetic variants must be strongly associated with the exposure of interest.^[2]^ The genetic variants should not be linked to any confounding factors.^[3]^ The genetic variants should influence the outcome only through the exposure, ensuring the association is not due to direct effects.

Our analysis exclusively utilized publicly available summary-level data from extensive GWAS and consortia, eliminating the need for additional ethical approval. All primary studies included in our research had already received approval from appropriate ethics committees, with informed consent obtained from participants. We followed the strengthening the reporting of observational studies in epidemiology for Mendelian randomization guidelines to ensure transparency and methodological rigor.^[14,15]^

### 2.1. GWAS data sources for hematological parameters

We utilized data predominantly from the UK Biobank consortium to ensure consistency in population base and temporal context. The PCa dataset (GWAS ID: ebi-a-GCST90018905, 2021) included 11,599 cases and 1,99,628 controls, all of European descent. Similarly, data for hematological traits were drawn from the same source to maintain uniformity. For example, the white blood cell count dataset (GWAS ID: ebi-a-GCST90018978, 2021) comprised 3,50,470 participants. A complete summary of all datasets used, including sample sizes and key variables, is provided in Table S1, Supplemental Digital Content, https://links.lww.com/MD/Q114.

To ensure broader genetic representation, the selected GWAS datasets were derived from a large-scale, cross-population genetic study encompassing 220 human phenotypes, integrating data from multiple biobanks, including UK Biobank, Biobank Japan, and FinnGen. This approach enhances the robustness and generalizability of our findings. Additionally, as the datasets originate from different biobanks, the probability of sample overlap is negligible, minimizing potential biases due to shared participants.

This study exclusively utilized publicly available summary-level data from previously published GWAS. All primary studies included had received approval from the relevant ethical review boards, and informed consent was obtained from participants. Therefore, no additional ethical approval was required.

### 2.2. Instrumental variables selection

**Figure 2.**
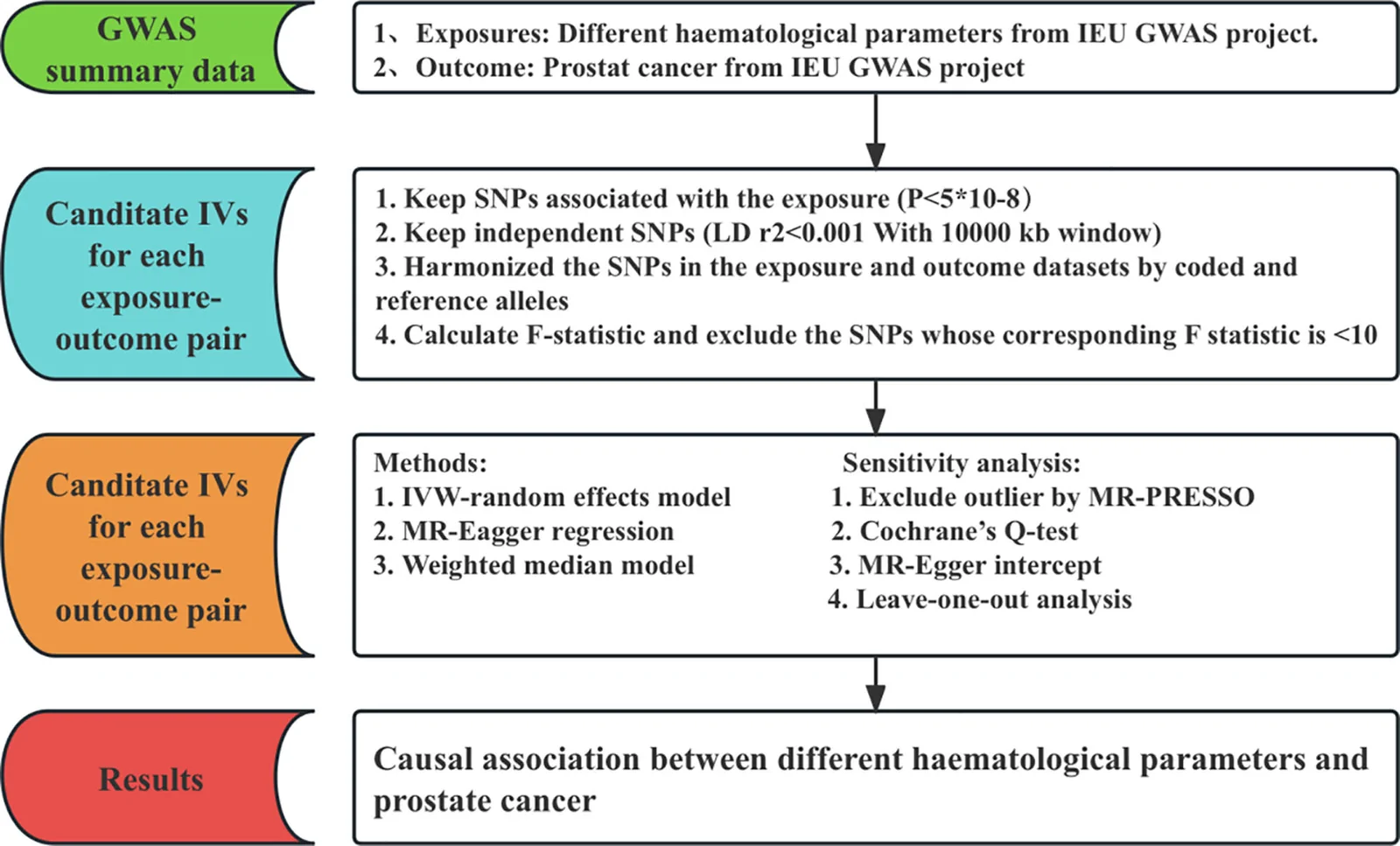
The schematic representation of inclusion and exclusion criteria for candidate SNPs in each exposure-outcome pairing, with a subsequent application of MR and the utilization of IVW methodology, is delineated in the flowchart. GWAS = genome-wide association studies, IEU = Integrative Epidemiology Unit, IV = instrumental variable, IVW = inverse variance weighted, MR = Mendelian randomization, MR-PRESSO = Mendelian randomization with pleiotropy residual sum and outlier detection, SNP = single nucleotide polymorphisms.

To ensure data integrity, we applied strict filtering criteria, excluding SNPs with high linkage disequilibrium (*r*^2^ > 0.001, clumping window < 10,000 kb) and retaining only those with a *P*-value < 5 × 10⁻⁸. We aligned coded and reference alleles between exposure and outcome datasets, excluding palindromic SNPs with ambiguous allele frequencies to improve data fidelity. The impact of missing SNPs was minimal, so proxy SNPs were not required. Instrument strength was assessed using the *F*-statistic, with values above 10 indicating robust instruments. To further reduce potential confounding, we used the PhenoScanner V2 database to identify and exclude SNPs associated with traits such as body mass index, Type II diabetes, alcohol consumption, LDL cholesterol levels, smoking, and other relevant factors. Additionally, we manually reviewed SNP associations to ensure no strong pleiotropic effects, beyond those detectable by statistical tests. A detailed schematic of the methodology is provided in Figure 2.

### 2.3. Statistical analysis

We conducted bidirectional univariable MR analyses to explore causal relationships between hematological parameters and PCa. SNPs associated with the exposures were selected from GWAS, with careful allele alignment to ensure consistency between exposure and outcome datasets, enhancing the precision of our findings.

Three MR methods were employed – inverse variance weighted (IVW), MR-Egger, and weighted median. IVW, our primary method, assumes valid IVs and aggregates their effects to estimate the causal impact of the exposure. MR-Egger adjusts for horizontal pleiotropy, identifying potential biases through its regression intercept, though with reduced statistical power. The Weighted Median method derives robust causal estimates by prioritizing variants based on their strength of association with the exposure, ensuring resilience against violations of IV assumptions. We excluded additional methods such as Simple Mode and Weighted Mode due to their minimal impact on the results and the large number of exposure factors analyzed.

Statistically significant exposures identified in univariable MR analyses were further evaluated using multivariable MR analysis. This approach allows independent assessment of multiple factors, similar to evaluating treatments in randomized trials. Unlike traditional MR methods focused on single risk factors, our strategy accommodates instruments linked to multiple exposures, providing a comprehensive view of causal relationships.

To ensure robustness, we performed sensitivity analyses using Mendelian randomization with pleiotropy residual sum and outlier detection (MR-PRESSO), MR-Egger intercept tests, and Cochran’s *Q* test to assess pleiotropy and heterogeneity. MR-PRESSO was employed to detect and correct outlier SNPs that might introduce pleiotropy, and Cochran’s *Q* test was used to quantify heterogeneity across instruments. When heterogeneity was detected (*P* < .05), a random-effects IVW model was applied. A leave-one-out analysis was also conducted to confirm that no single SNP disproportionately influenced the results. MR-PRESSO was employed to detect and correct outliers, further enhancing the reliability of our findings.

Statistical significance was set at *P* < .05, with significant findings from univariable MR considered indicative rather than conclusive. All analyses were performed using R (version 4.2.3), with packages such as ggplot2 (version 3.4.3), TwoSampleMR (version 0.5.7), and MR-PRESSO (version 1.0). These tools ensured precise statistical evaluations, minimizing redundancy and reinforcing the rigor and reliability of our study.

## 3. Results

Table S2, Supplemental Digital Content, https://links.lww.com/MD/Q115 provides key parameters for each phenotype, including heterogeneity test *P*-values, MR-Egger intercept *P*-values, and other metrics. All SNPs used in the analysis exhibited *F*-statistics >10, confirming strong IV strength and minimizing the influence of weak instruments. Moderate heterogeneity was detected in most exposure analyses (Cochran’s *Q P* < .05), necessitating the use of the random-effects IVW MR method. MR-PRESSO analysis identified global heterogeneity, indicating the potential presence of horizontal pleiotropy. However, after correcting for outliers, the impact of genotype on outcomes was no longer statistically significant, suggesting that pleiotropic effects were adequately mitigated. Additionally, MR-Egger regression analysis did not detect significant pleiotropy (all *P* > .05), further supporting the robustness of the IVs and the reliability of the causal inferences.

**Figure 3.**
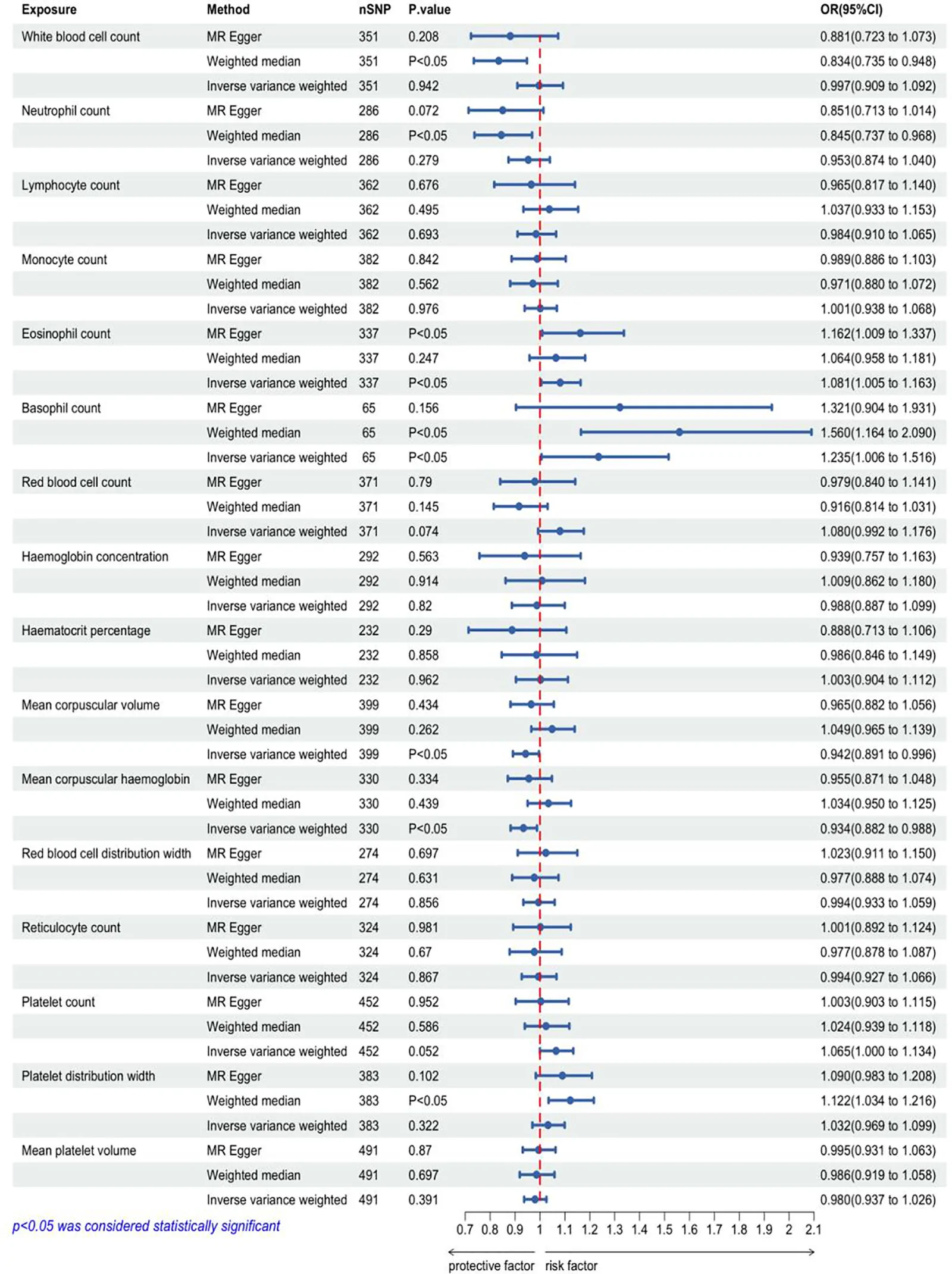
Univariable MR outcomes elucidating the impacts of diverse hematological parameters on PCa. CI = confidence interval, MR = Mendelian randomization, OR = odds ratio, SNP = single nucleotide polymorphisms.

**Figure 4.**
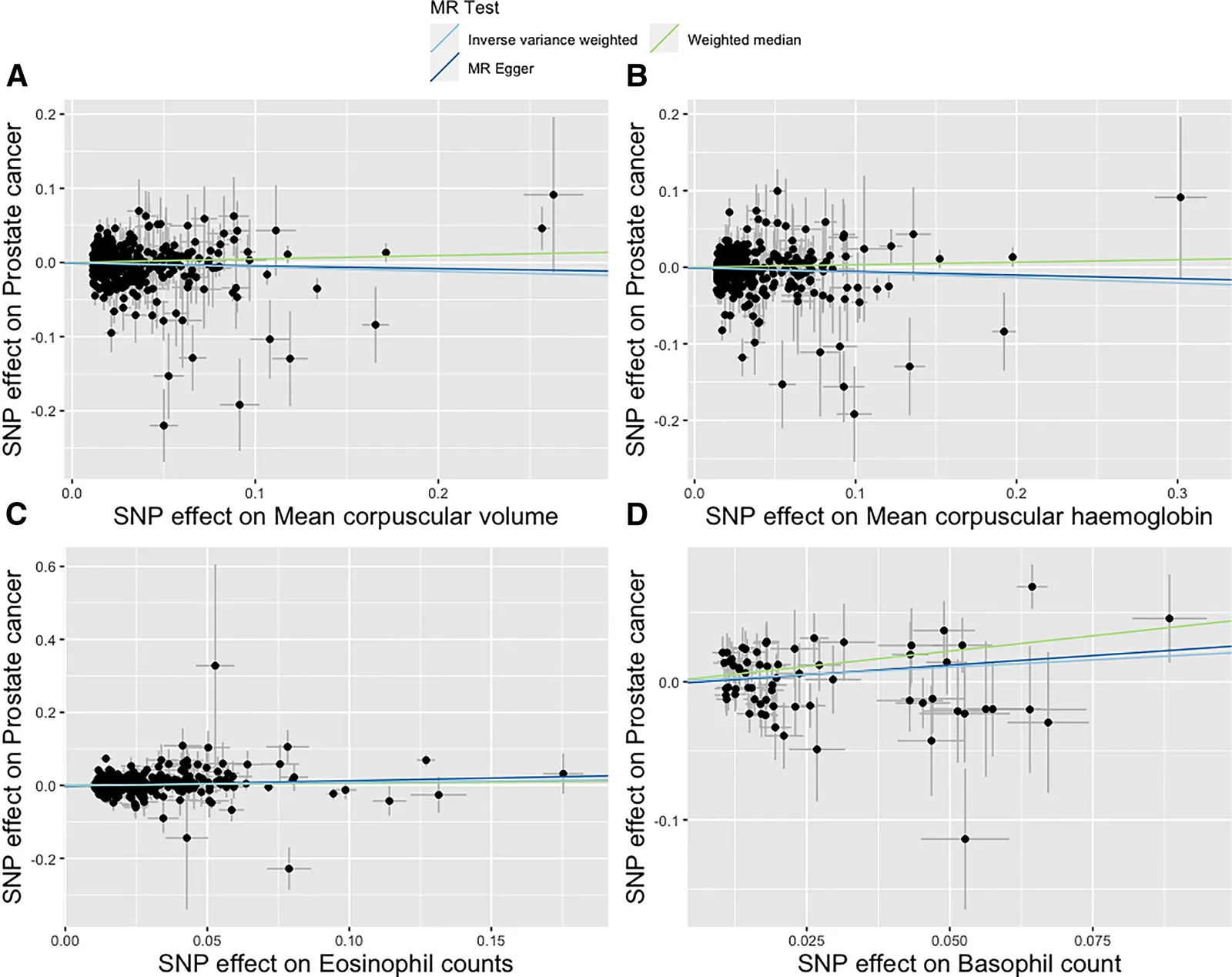
A scatter plot that demonstrates the relationships between MCV, MCH, eosinophil counts, basophil counts and PCa. MCH = mean corpuscular hemoglobin, MCV = mean corpuscular volume, MR = Mendelian randomization, PCa = prostate cancer, SNP = single nucleotide polymorphisms.

Figure 3 summarizes the results of the univariate MR analyses. Using the IVW approach, MCV (OR: 0.942, 95% CI: 0.891–0.996, *P* = .035) and MCH (OR: 0.934, 95% CI: 0.882–0.988, *P* = .018) were inversely associated with PCa risk, suggesting a protective effect. In contrast, higher eosinophil counts (OR: 1.081, 95% CI: 1.005–1.163, *P* = .036) and basophil counts (OR: 1.235, 95% CI: 1.006–1.516, *P* = .044) were associated with an increased risk of PCa. These relationships are visualized in the scatter plot of Figure 4. No significant associations were observed between PCa and other hematological parameters, such as white blood cell count, neutrophil count, lymphocyte count, monocyte count, RBC count, hemoglobin concentration, hematocrit percentage, RBC distribution width, reticulocyte count, platelet count, platelet distribution width, or mean platelet volume (all *P* > .05).

**Figure 5.**
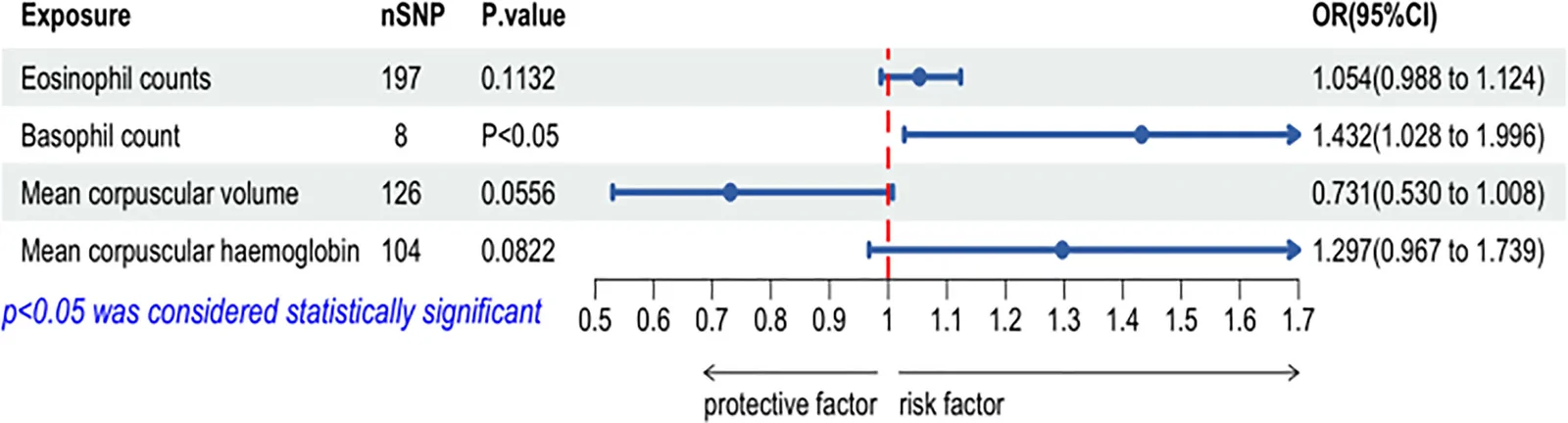
Multivariable MR findings delineating the effects of eosinophil counts, basophil counts, mean corpuscular volume, and mean corpuscular hemoglobin on PCa. CI = confidence interval, MR = Mendelian randomization, OR = odds ratio, PCa = prostate cancer, SNP = single nucleotide polymorphisms.

In the multivariable MR analysis that included MCV, MCH, eosinophil count, and basophil count, only basophil count showed a significant association with PCa (OR: 1.432, 95% CI: 1.028–1.996, *P* = .034; Fig. 5). The associations for MCV, MCH, and eosinophil count were weaker, with all *P*-values exceeding .05.

**Figure 6.**
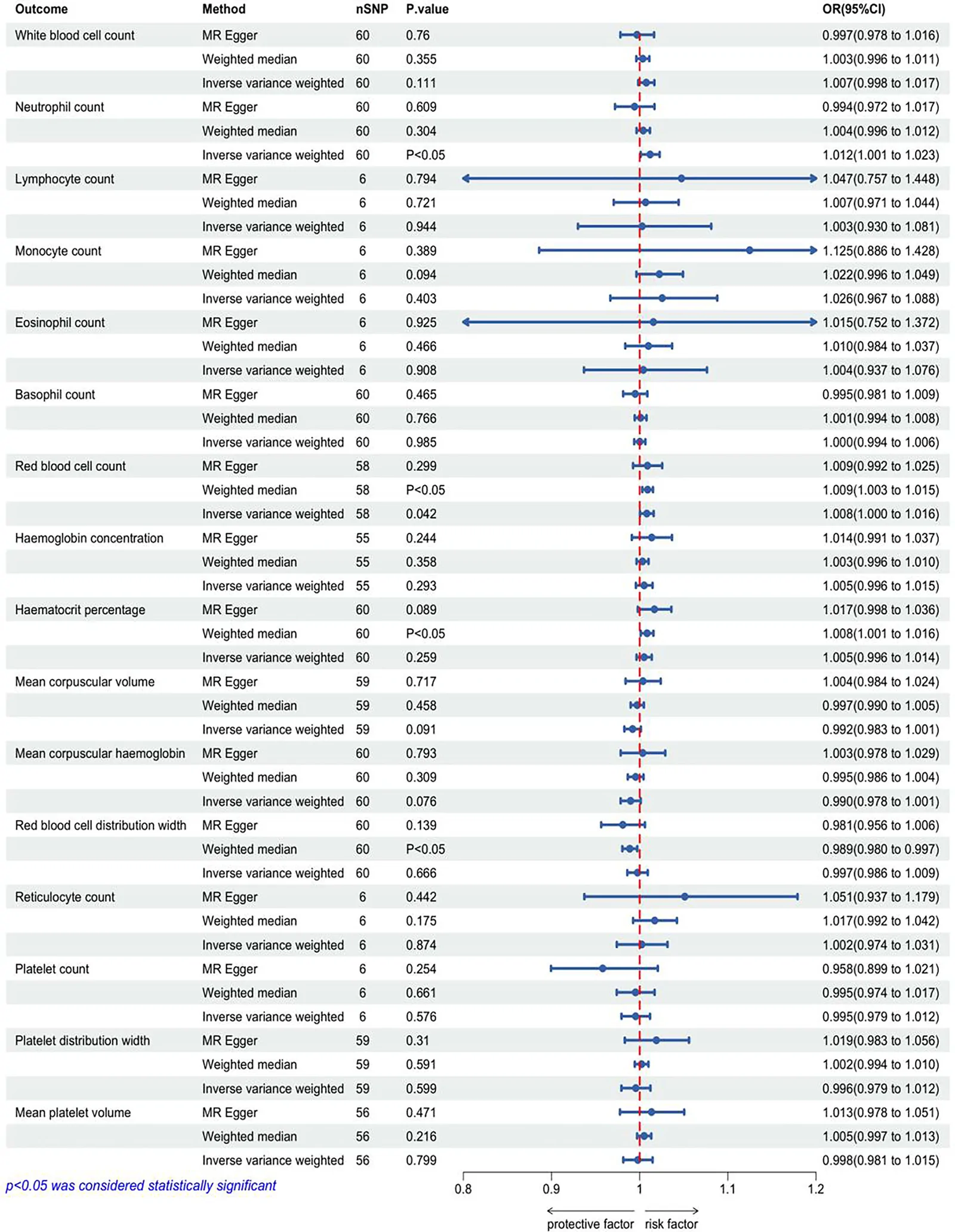
Univariable Mendelian randomization findings illustrating the impact of PCa on various hematological parameters. CI = confidence interval, MR = Mendelian randomization, OR = odds ratio, PCa = prostate cancer, SNP = single nucleotide polymorphisms.

**Figure 7.**
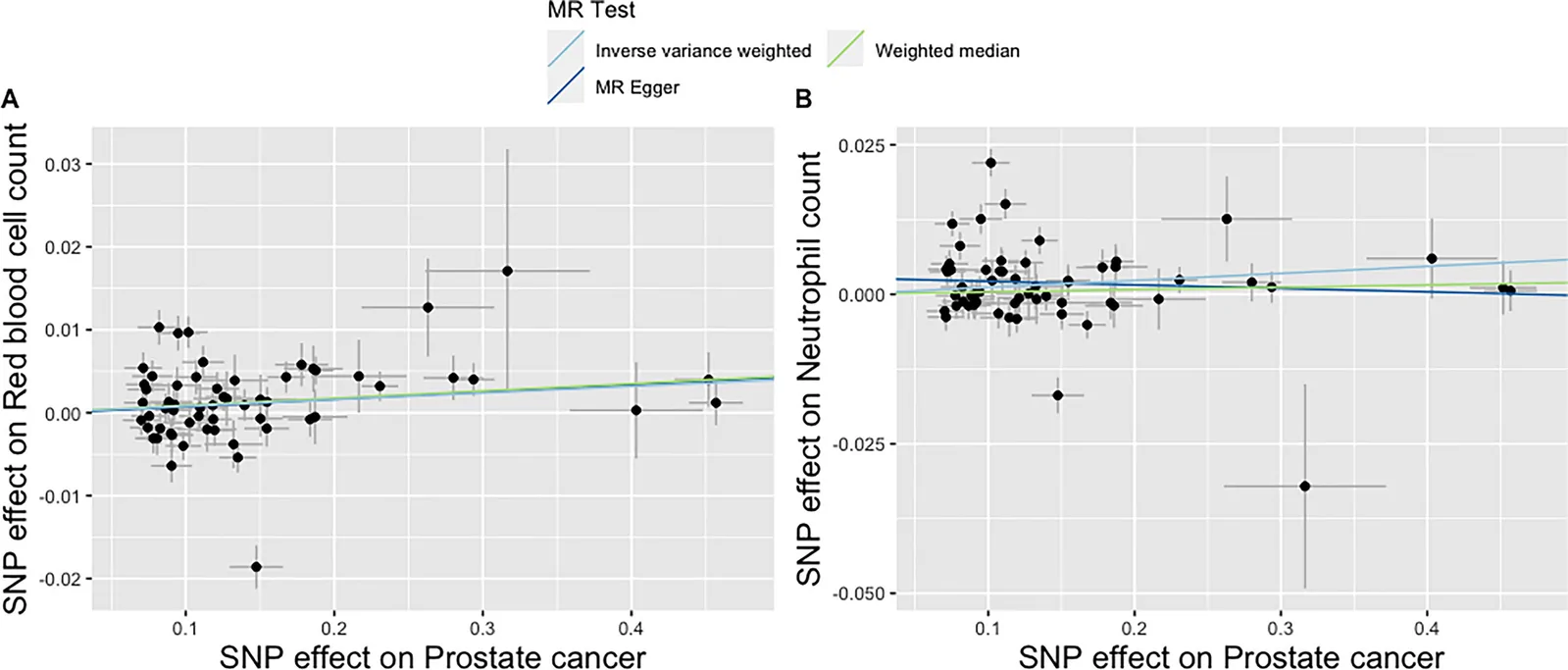
A scatter plot that illustrates the relationships between PCa and red blood cell count as well as neutrophil count. MR = Mendelian randomization, PCa = prostate cancer, SNP = single nucleotide polymorphisms.

A reverse MR analysis was conducted to assess the potential impact of PCa on hematological parameters (Fig. 6). The analysis revealed that PCa may lead to an increase in neutrophil count (OR: 1.012, 95% CI: 1.001–1.023, *P* = .031) and RBC (OR: 1.008, 95% CI: 1.000–1.016, *P* = .042). These findings are depicted in the scatter plot of Figure 7.

## 4. Discussion

In this study, univariate MR analyses revealed an inverse association between MCV or MCH and PCa, while eosinophil and basophil counts were positively associated with PCa risk. However, no significant causal relationships were found for other hematological parameters. Multivariable MR analysis further identified elevated basophil count as significantly associated with increased PCa incidence, whereas MCV, MCH, and eosinophil counts exhibited weaker associations. Reverse MR analysis suggested that PCa may lead to increased neutrophil and RBCs, underscoring the intricate relationship between hematological parameters and PCa and the need for further research to clarify these associations. These findings suggest that hematological markers may have potential prognostic value in PCa. Neutrophil elevation, commonly linked to systemic inflammation, aligns with the neutrophil-to-lymphocyte ratio, a known predictor of poor PCa outcomes. Similarly, increased RBCs may indicate metabolic changes associated with advanced disease. Monitoring these parameters could help stratify patients at higher risk for aggressive PCa and refine prognostic models, warranting further clinical validation.

Our findings align partially with prior observational studies exploring the relationship between hematologic markers and PCa. For instance, Watts et al., using UK Biobank data, identified higher RBC and platelet counts as PCa risk factors, while increased MCV, MCH, and MCH concentration were associated with reduced risk.^[6]^ Our results corroborate some of these findings, particularly through reverse MR analysis, which linked elevated neutrophil count to PCa, possibly contributing to increased PCa mortality. However, we did not observe a significant causal association between platelet count and PCa. This may be due to differences between MR and observational studies, as MR reduces confounding and reverse causation. Further research is needed to clarify the potential role of platelets in PCa development. Similarly, research by Porcaro AB et al identified white blood cell count as a predictor of PCa risk among patients undergoing transurethral resection of the prostate.^[16]^ Although our study did not replicate this finding, it highlights the potential relevance of hematologic parameters as risk indicators. Research by Bahig et al has associated neutrophil count with overall survival in localized PCa,^[9]^ Our findings align with this observation, further emphasizing the need for future studies to explore the prognostic role of neutrophil count. Additionally, Hadadi et al found a link between elevated basophil count and worse outcomes in metastatic hormone-sensitive PCa.^[10]^ Our data corroborate this finding, reinforcing the potential of basophil count as a biomarker for both PCa risk and prognosis. Collectively, these studies and our results highlight the complex interplay between hematological markers and PCa, emphasizing the importance of further exploration to fully understand the clinical and biological implications.

Previous studies have also identified associations between hematologic markers and PCa progression. Wang et al reported that preoperative red cell distribution width was associated with lymphovascular invasion in PCa, suggesting a role in disease advancement.^[17]^ Albayrak et al found that red cell distribution width predicted increased PCa risk, further supporting its importance in disease progression.^[18]^ Similarly, Yu et al linked elevated platelet counts to poorer outcomes in PCa.^[19]^ Song et al proposed that integrating platelet distribution width with PSA levels could enhance diagnostic precision.^[20]^ However, our study did not replicate these associations, possibly reflecting the influence of confounding variables or complex interactions between hematological markers and PCa. These discrepancies underscore the need for careful interpretation of hematologic indicators and further research to delineate their roles in PCa pathophysiology and clinical management.

From a clinical perspective, hematological markers such as MCV, MCH, eosinophils, and basophils are easily accessible and cost-effective compared to traditional PCa biomarkers like PSA. However, rather than replacing PSA, these markers could serve as complementary tools to refine risk stratification and improve screening accuracy. For instance, individuals with elevated basophil counts may represent a high-risk subgroup that warrants closer monitoring, while higher MCV or MCH levels could indicate a lower-risk profile, potentially helping to reduce unnecessary biopsies in borderline PSA cases. By integrating hematological parameters with established risk models, clinicians may be able to enhance early detection strategies and optimize patient management.

Unraveling the complex relationship between hematological markers and PCa remains challenging, as few definitive factors have been identified to drive PCa initiation and progression. Our findings suggest that MCV and MCH may have protective effects, potentially reflecting metabolic activities that inhibit tumor growth. One possible explanation is that increased RBC volume and hemoglobin content may enhance oxygen delivery, reducing hypoxia-induced tumor progression. Hypoxia is a well-established driver of aggressive tumor phenotypes through the activation of hypoxia-inducible factors (HIF-1α), which promote angiogenesis, metabolic reprogramming, and immune evasion.^[21]^ Previous studies have suggested that altered erythropoiesis in the tumor microenvironment could promote hypoxia-driven cancer cell adaptation and tumor progression, which contrasts with our findings that RBC indices may act as protective factors against PCa progression.^[22]^ However, given the complexity of these interactions, further research is needed to comprehensively evaluate the role of RBC metrics in PCa.

Conversely, increased eosinophil and basophil counts may indicate heightened inflammatory responses, which have been linked to cancer risk. Chronic inflammation plays a crucial role in tumor microenvironment dynamics, immune surveillance, and cancer progression through pathways involving chemokines and cytokines.^[23,24]^ The observed elevations in eosinophil and basophil counts may thus promote PCa progression through inflammation-related mechanisms.^[25,26]^ Basophils, for instance, release histamine, interleukins (IL; e.g., IL-4, IL-13), and other pro-inflammatory mediators that contribute to an immunosuppressive tumor microenvironment, facilitating immune escape and promoting tumor proliferation.^[27]^ Similarly, eosinophils have been implicated in both tumor-promoting and antitumor activities, depending on the context.^[28,29]^ Studies have shown that eosinophils can enhance tumor-associated inflammation by interacting with regulatory T cells and secreting transforming growth factor-β, which promotes immune tolerance and cancer progression.^[28]^ However, our study expands on this conclusion, suggesting that eosinophils serve as a risk factor for PCa. Given these findings, hematological markers could serve not only as diagnostic indicators but also as potential therapeutic targets. Identifying whether anti-inflammatory interventions targeting eosinophils or basophils could modify PCa progression is a promising area for future clinical research.

Furthermore, our reverse MR analysis indicated that PCa may increase both neutrophil and RBCs. Elevated neutrophil counts may reflect inflammation-induced metabolic shifts, while increased RBCs could be associated with PCa’s proclivity for bone marrow metastasis. Bone marrow stromal cells, known to support PCa cell survival by inhibiting apoptosis-inducing signals, may contribute to increased blood cell production.^[30]^ Additionally, as tumors progress, their oxygen consumption increases,^[31]^ potentially driving higher levels of oxygen-carrying RBCs. In renal cell carcinoma, tumor hypoxia, primarily via HIF-2α activation, induces erythropoietin secretion, stimulating erythropoiesis. This effect is particularly evident in VHL-deficient renal cell carcinoma, where increased erythropoietin contributes to elevated RBC levels.^[32]^ The presence of a similar feedback mechanism in PCa warrants further investigation.

Our findings elucidate potential causal relationships between PCa and several hematological markers, including MCV, MCH, eosinophil count, basophil count, neutrophil count, and RBC. While these associations suggest biological mechanisms, further research is needed to clarify the underlying principles and confirm these observations. Notably, the significant association between elevated basophil count and PCa incidence observed in our multivariable MR analysis is a novel finding, signaling a new direction for future research into PCa pathophysiology.

This study leverages state-of-the-art genetic tools and extensive GWAS datasets to explore the causal links between hematological markers and PCa. Our bidirectional and multivariable MR approach, combined with comprehensive sensitivity analyses, ensures the robustness of our findings and minimizes pleiotropic bias. We specifically focused on individuals of European ancestry to reduce population stratification bias. Consistency across multiple data sources and MR models reinforces the credibility of our results, suggesting minimal influence from horizontal pleiotropy.

Nevertheless, several limitations should be acknowledged. Cochran’s *Q* test revealed heterogeneity among IVs, necessitating the use of the IVW random-effects model for its robustness.^[12]^ MR-PRESSO identified and corrected outliers, reducing the impact of influential values. Importantly, sensitivity analyses demonstrated that our primary findings remained stable across different MR methods, suggesting that heterogeneity did not significantly undermine the validity of our conclusions. The observed heterogeneity may arise from variations in genetic instruments, differences in GWAS cohorts, or potential residual confounding. However, our stringent SNP selection criteria, exclusion of pleiotropic variants, and use of multiple sensitivity tests mitigate these concerns. Furthermore, leveraging large-scale GWAS data enhances the generalizability and statistical power of our findings. To further improve the robustness and applicability of these associations, future studies should incorporate more diverse populations using multiethnic GWAS datasets and trans-ethnic MR approaches. Expanding genetic investigations beyond European cohorts will be crucial for refining risk prediction models and improving their clinical relevance. Additionally, refining IV selection and incorporating advanced statistical methods may help minimize residual heterogeneity and strengthen causal inferences.

## 5. Conclusion

In conclusion, our MR analysis reveals that MCV and MCH are associated with reduced PCa risk, while elevated eosinophil and basophil counts increase risk. Reverse MR suggests that PCa may elevate neutrophil and RBCs, indicating possible feedback mechanisms. These findings enhance understanding of the genetic links between hematological markers and PCa, but further research is needed to confirm these associations and explore their underlying mechanisms.

## Acknowledgments

We wish to acknowledge the participants and investigators of the Integrative Epidemiology Unit (IEU, https://gwas.mrcieu.ac.uk/).

## Author contributions

**Conceptualization:** Yirui Wei.

**Data curation:** Yirui Wei, Liang Wang, Pushen Yang, Dawei Xie.

**Formal analysis:** Yirui Wei, Dawei Xie.

**Funding acquisition:** Yirui Wei, Jianwen Wang.

**Investigation:** Yirui Wei, Liang Wang, Weifeng He, Jianwen Wang.

**Methodology:** Yirui Wei, Hao Wang, Liang Wang.

**Project administration:** Yirui Wei.

**Resources:** Yirui Wei, Hao Wang, Weifeng He.

**Software:** Yirui Wei, Hao Wang, Pushen Yang.

**Supervision:** Yirui Wei, Hao Wang, Pushen Yang, Jianwen Wang.

**Validation:** Yirui Wei, Jianwen Wang.

**Visualization:** Yirui Wei, Hao Wang, Pushen Yang, Jianwen Wang.

**Writing – original draft:** Yirui Wei, Hao Wang, Liang Wang.

**Writing – review & editing:** Yirui Wei, Hao Wang, Liang Wang.

## Supplementary Material
